# Supplementary material for: Accurate Prediction of Cancer Prognosis by Exploiting Patient-Specific Cancer Driver Genes
Source: Int J Mol Sci. 2023 Mar 29;24(7):6445. doi: 10.3390/ijms24076445 (PMC10095073; doi:10.3390/ijms24076445)

Figure S1. Comparison of various performance evaluation indeicators from 10 independent tests for each cancer.

S1 a. Comparison of average the Precision-Recall curve AUC

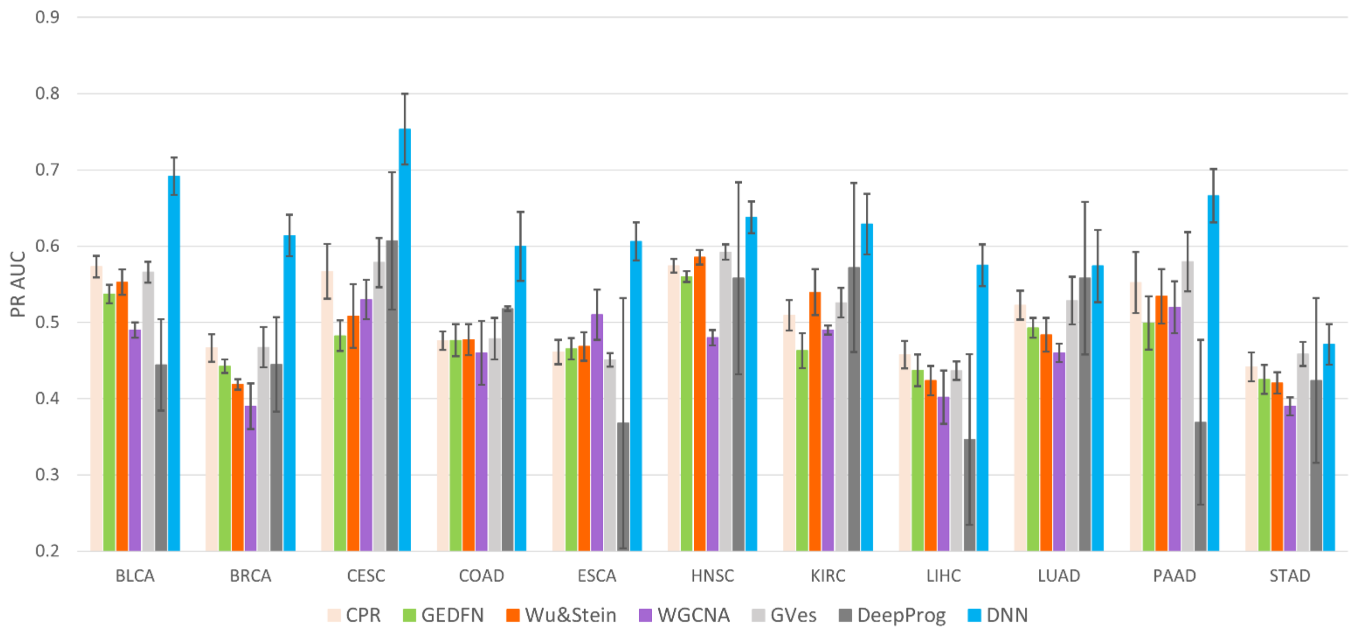

S1 b. Comparison of average balanced Accuracy

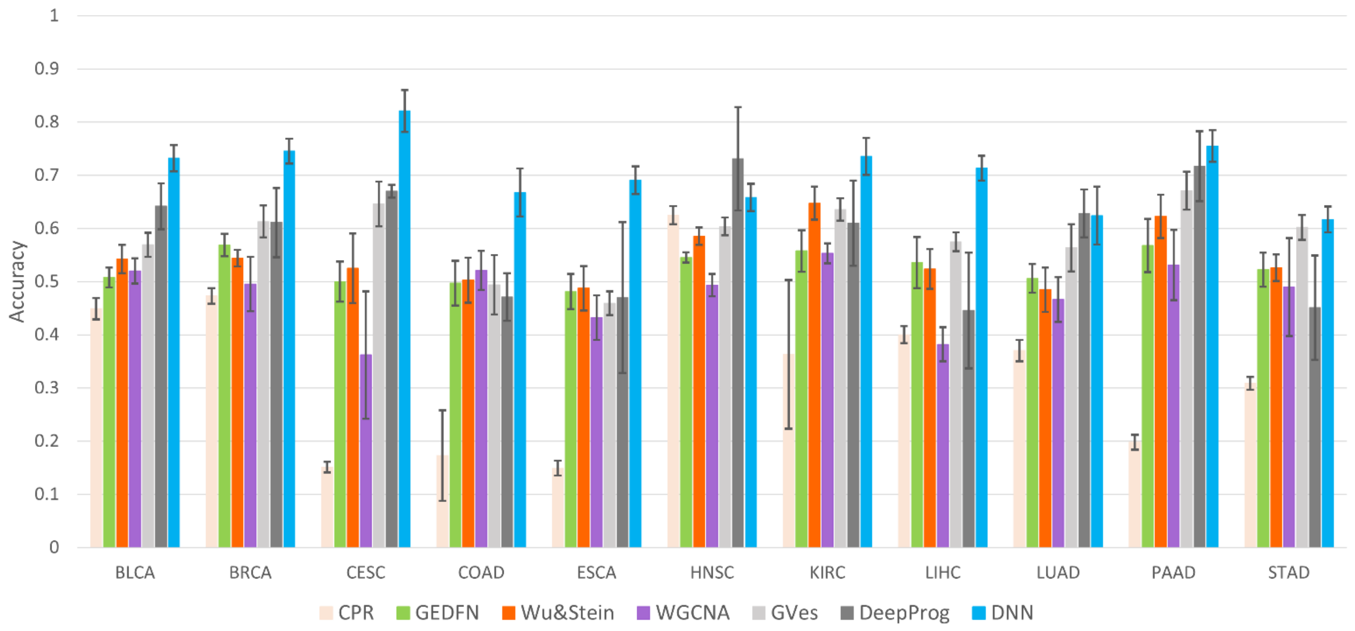

S1 c. Comparison of average F1-Score

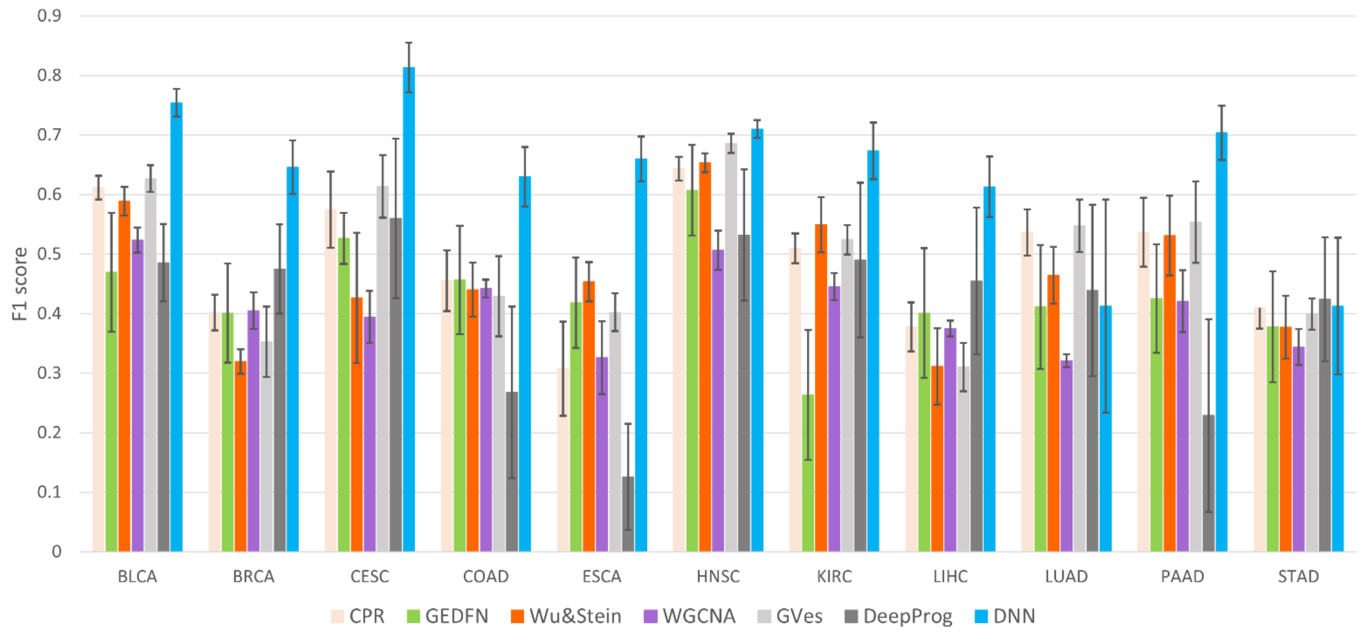

S1 d. Comparison of average Matthews Correlation Coefficient

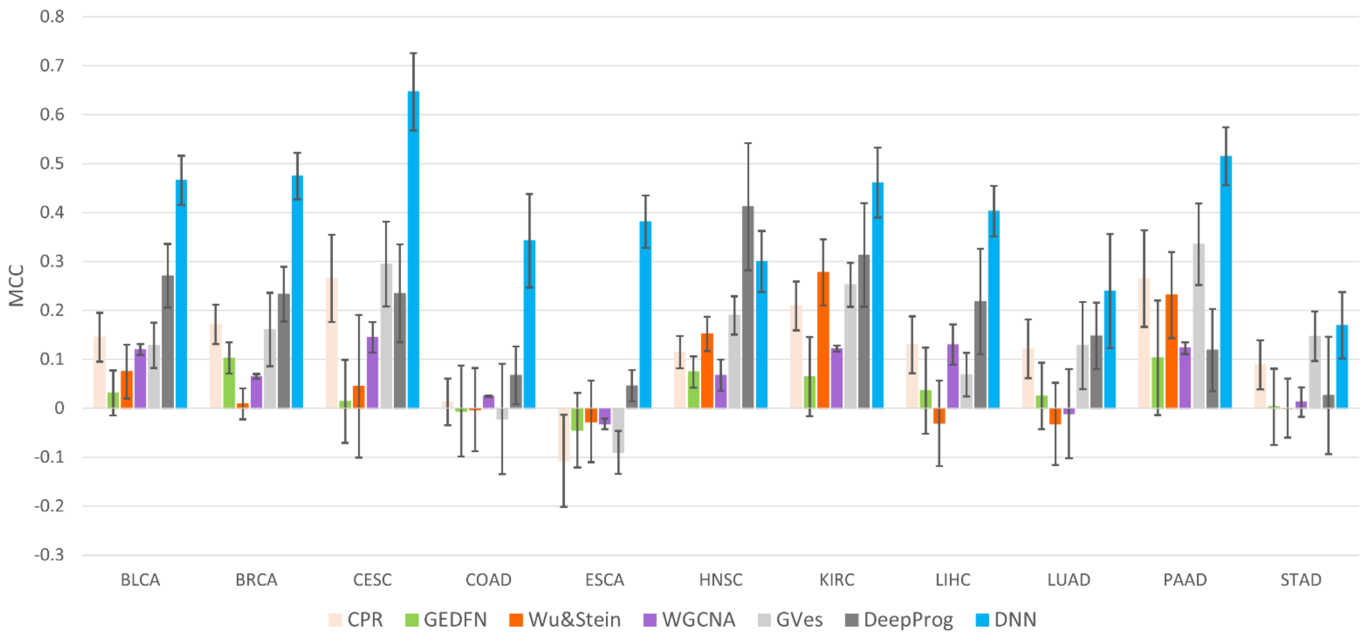

Figure S2. Breast cancer genes with p-value < 0.05 in performing Kaplan Meyer analysis.

S2 a. High-expressed gene group showing low survival probability

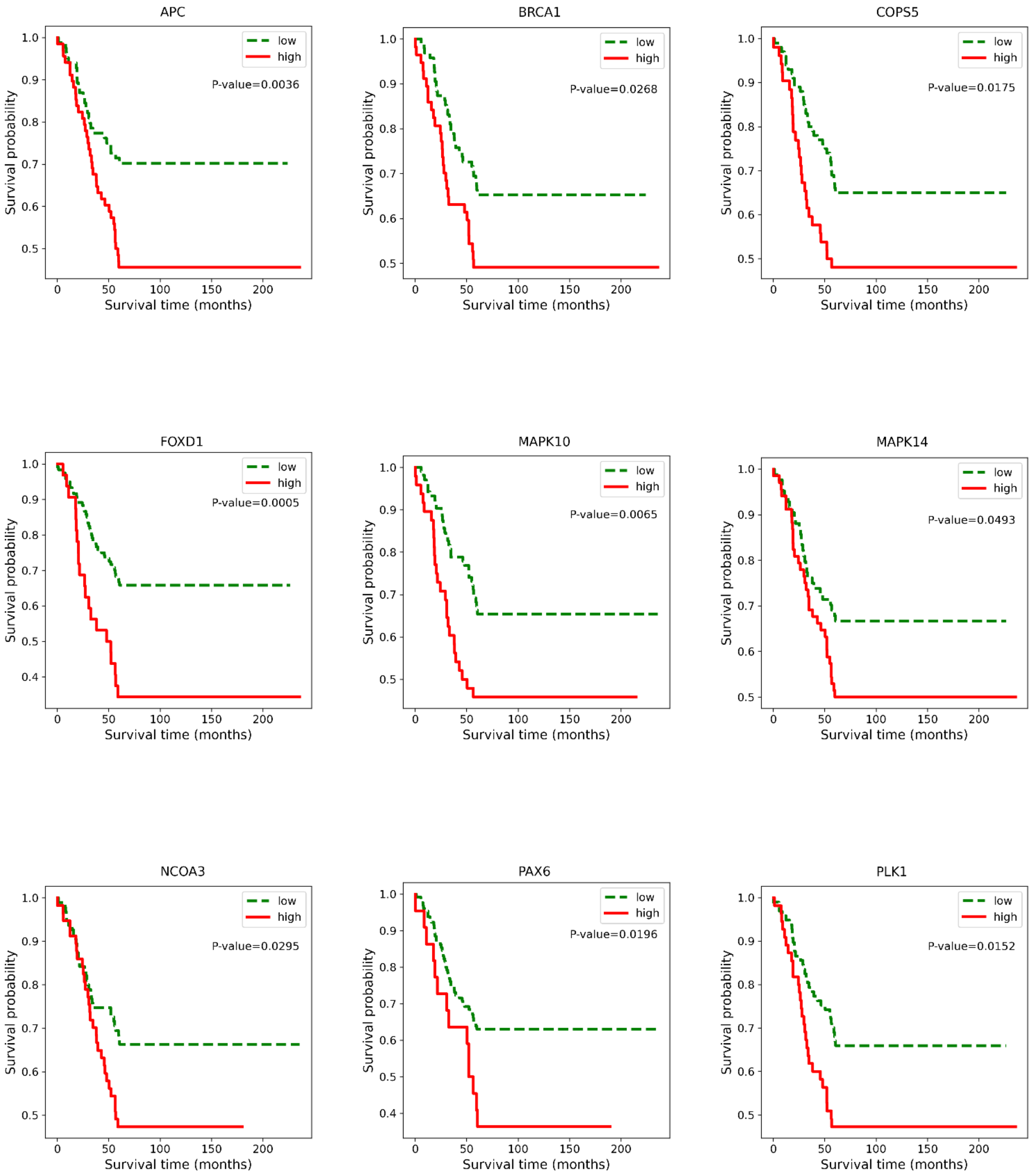

Figure S2. Breast cancer genes with p-value < 0.05 in performing Kaplan Meyer analysis.

S2 b. High-expressed gene group showing high survival probability

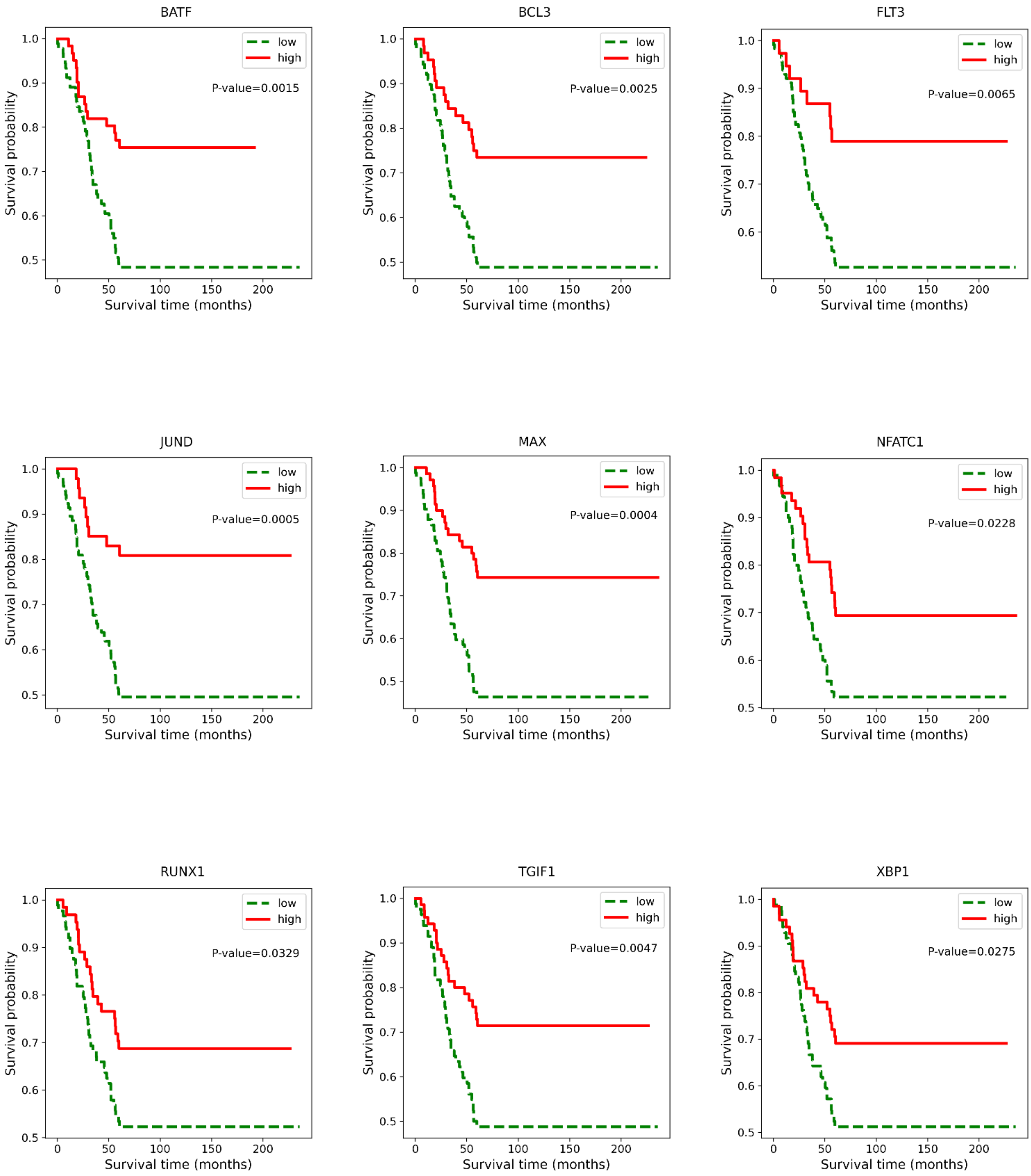

Figure S3. Breast cancer genes with p-value < 0.05 in performing Kaplan Meyer analysis using KM ploatter web-site.

S3 a. High-expressed gene group showing low survival probability

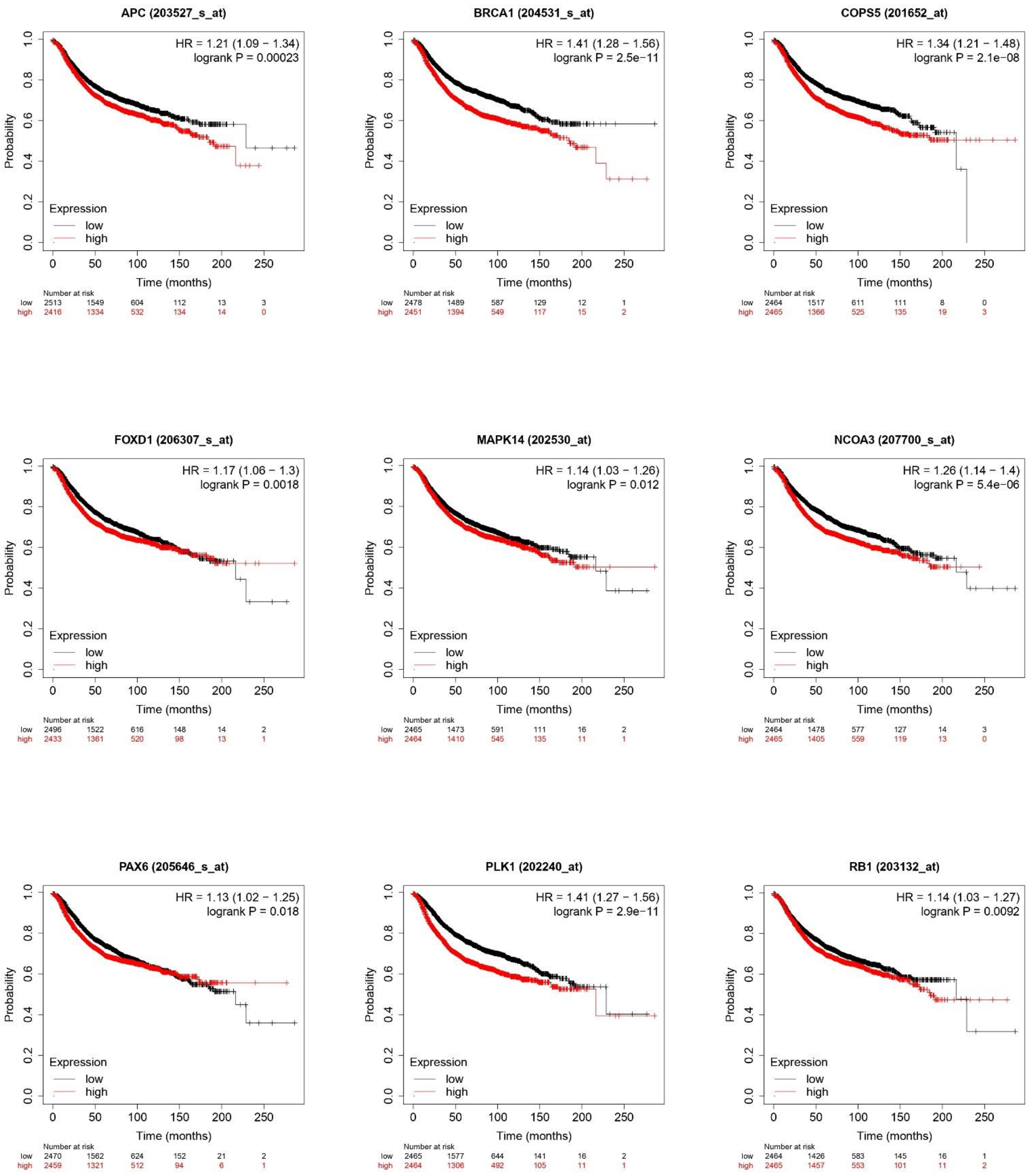

Figure S3. Breast cancer genes with p-value < 0.05 in performing Kaplan Meyer analysis using KM plotter web-site.

S3 b. High-expressed gene group showing high survival probability

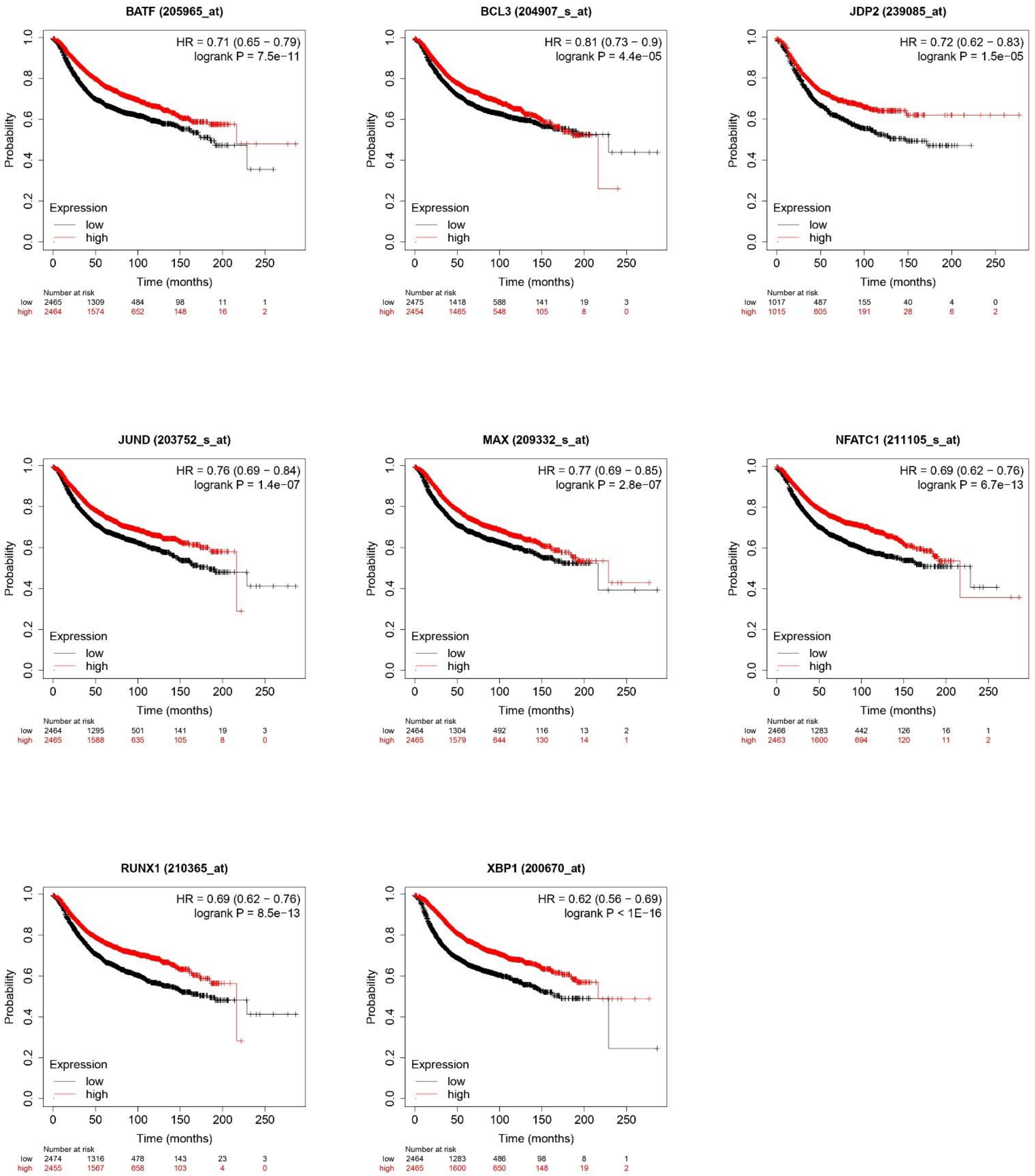

Figure S4. The correlation table of gene expression of breast cancer's genes with immune infiltration level in breast cancer types.

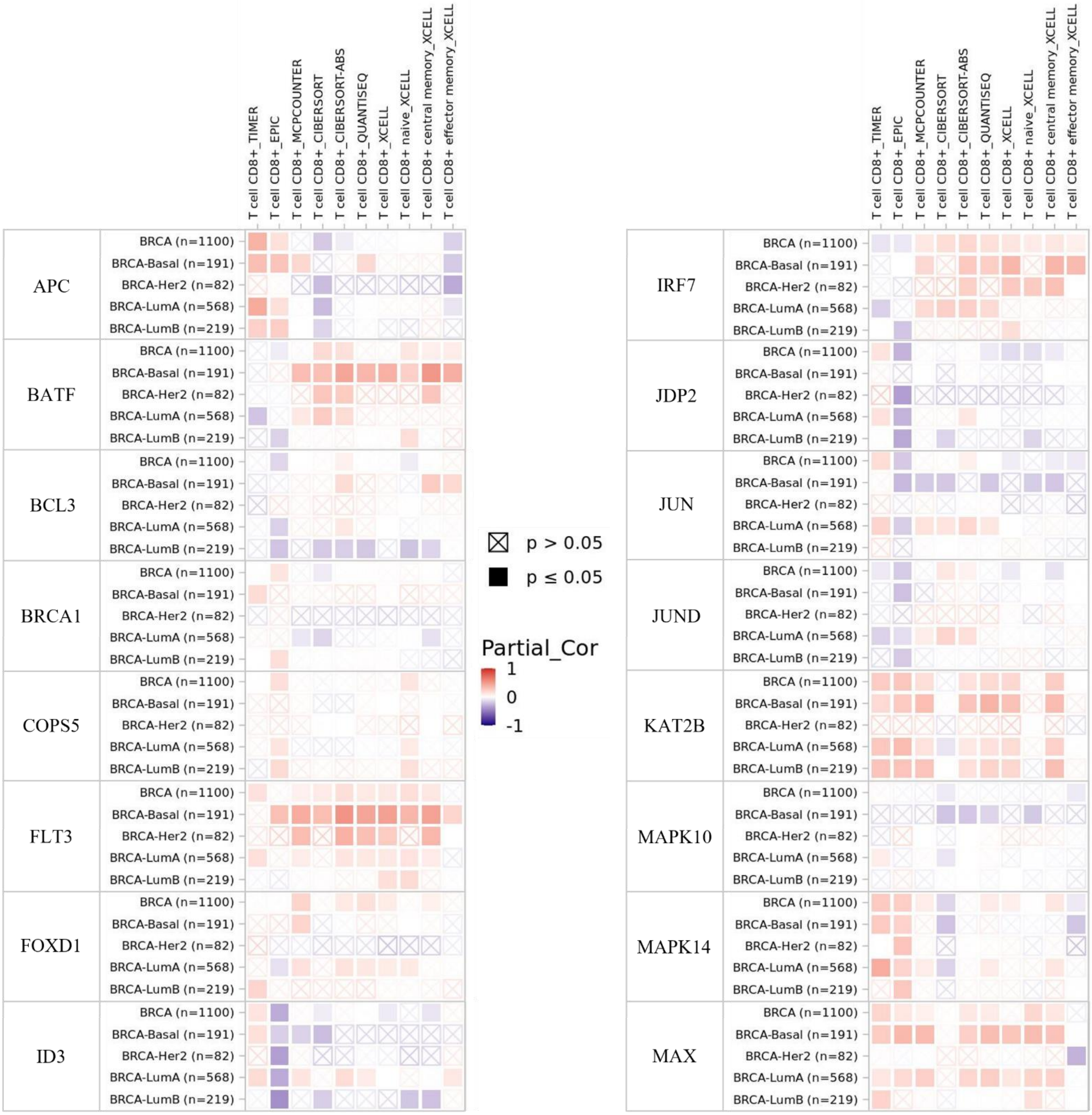

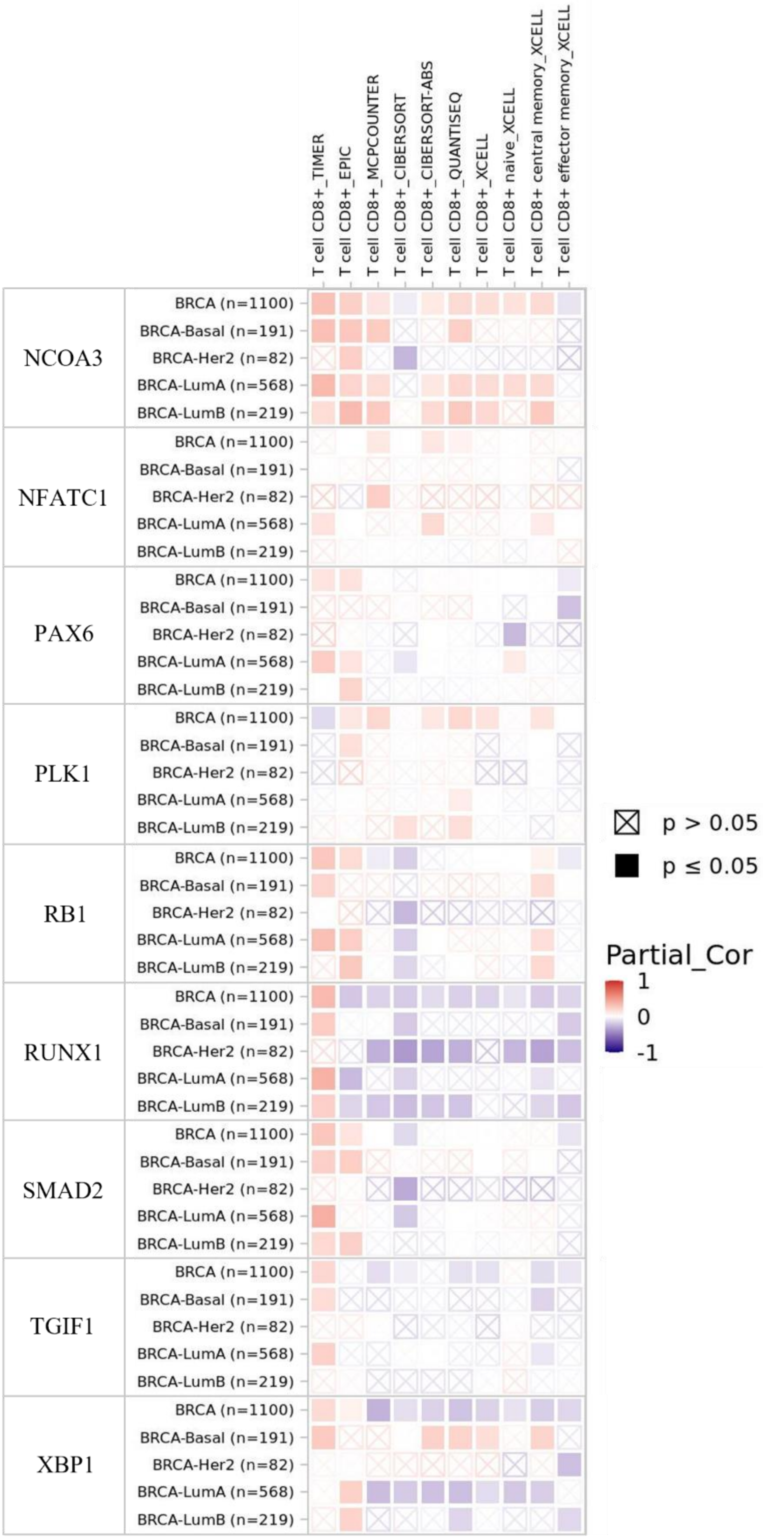
T cell CD8+_TIMER
T cell CD8+_EPIC
T cell CD8+_MCPCOUNTER
T cell CD8+_CIBERSORT
T cell CD8+_CIBERSORT-ABS
T cell CD8+_QUANTISEQ
T cell CD8+_XCELL
T cell CD8+ naive_XCELL
T cell CD8+ central memory_XCELL
T cell CD8+ effector memory_XCELL
NCOA3
NFATC1
PAX6
PLK1
RB1
RUNX1
SMAD2
TGIF1
XBP1
BRCA (n=1100)
BRCA-Basal (n=191)
BRCA-Her2 (n=82)
BRCA-LumA (n=568)
BRCA-LumB (n=219)
p > 0.05
p ≤ 0.05
Partial_Cor
1
0
-1

Figure S5. Cervical cancer genes with p-value < 0.05 in performing Kaplan Meyer analysis.

S5 a. High-expressed gene group showing low survival probability

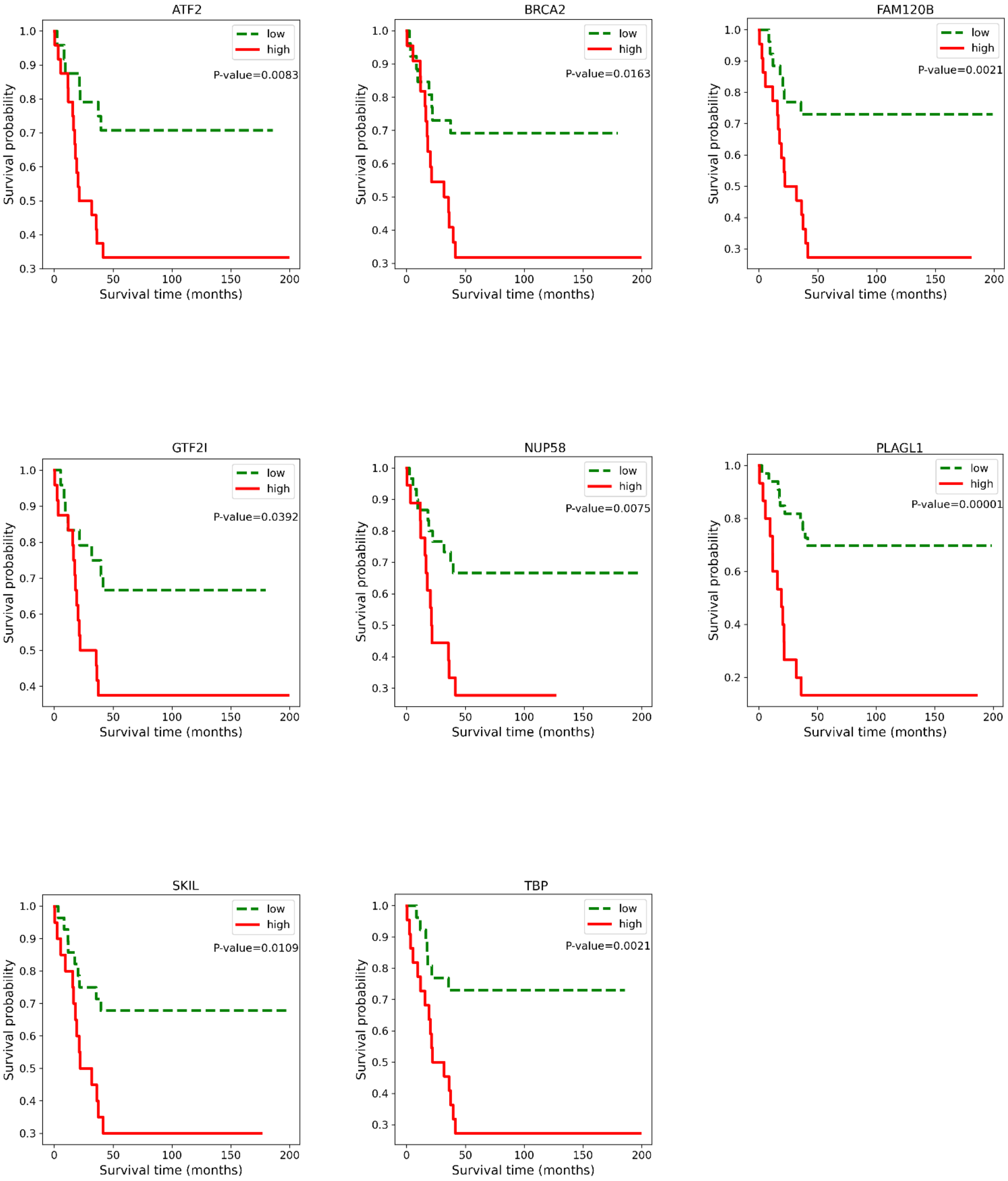

Figure S5. Cervical cancer genes with p-value < 0.05 in performing Kaplan Meyer analysis.

S5 b. High-expressed gene group showing high survival probability

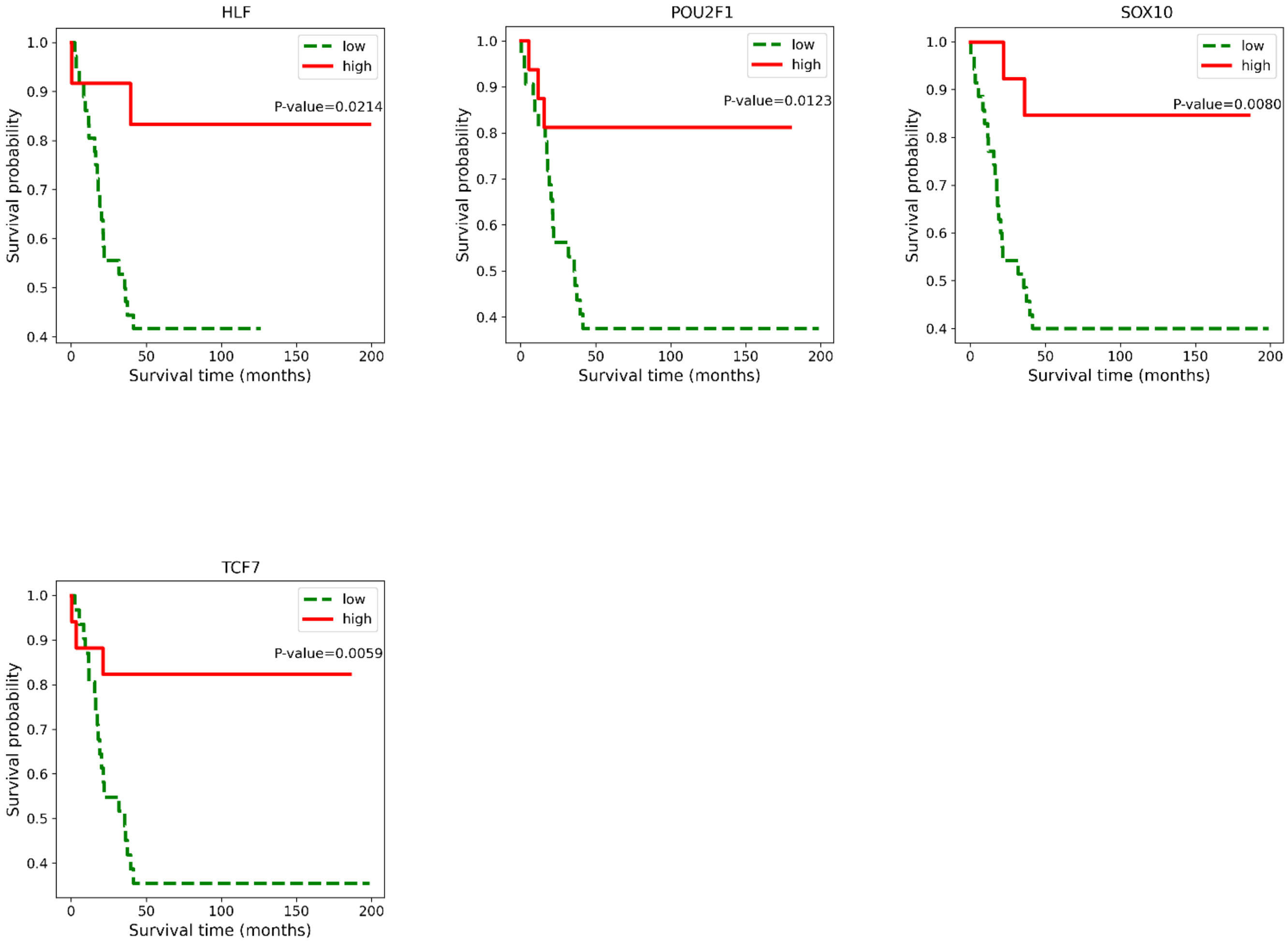

Supplement: Supplementary file 1 [file ijms-24-06445-s001.zip › Supplementary Figures.pdf]
